# 

**Published:** 1983

**Authors:** 


JANUARY/APRIL 1983
VOLUME 98 (i)
NUMBER 365
Published Quarterly
Annual Subscription £8 Post Free

### BRISTOL MEDICO-CHIRURGICAL JOURNAL

Contents

1. Editorial

Established 1883

### BRISTOL MEDICO-CHIRURGICAL JOURNAL

Incorporating the Medical Journal of the South West

### Editorial Committee

**EDITOR**
*Mr. M. G. Wilson*
*Litfield House, Clifton, Bristol BS8 3LS*

ASSISTANT EDITOR
*Dr. G. R. Tribe*
*Southmead Hospital, Bristol BS10 5NB*

MEMBERS
*Dr. M. B. Lennard*
*Dr. D. W. Wright*
*The Hon. Treasurer of the Society*
*The Hon. Secretary of the Society*

SECRETARY
*Mr. B. P. Jones, The Library, Medical School, University Walk, Bristol BS8 1TD*

CORRESPONDENTS
*Dr. Joseph Jancar*
*Dr. Gordon Mather*
*Professor Gordon Stirret*
*Dr. Gordon Thomson*
*Dr. Colin Tribe*
*Dr. Michael Whitfield*
*Professor Robin Williamson*
*Dr. Dennis Wright*

### Bristol Medico-Chirurgical Society

PRESIDENT 1980/81
*Mr. Herbert Bourns*

PRESIDENT ELECT
*Dr. Norman Tricks*

HONORARY SECRETARY
*Dr. H. M. Norman*
*Hillside, Vicarage Lane, Barrow Guerney*

HONORARY TREASURER
*Dr. J. Penry*
*Southmead Hospital, Bristol BS10 5NB*

The Society was founded in 1874. In 1883 publication of the Journal began and has continued quarterly ever since then. During the years 1949 to 1962 it appeared under the title of 'The Medical Journal of the South West'.

### Notice to Contributors

*The Journal is especially concerned to provide a place for the recording of medical thought, interests, and practice in and around Bristol. It therefore welcomes articles that, as well as being of immediate interest to members, will document the local and contemporary medical scene for our successors.*

*Original articles are invited provided that they have not been published elsewhere. Illustrations should be supplied ready for reproduction. Line drawings should be in Indian ink on firm white paper or board. The author will be informed if it is found necessary to ask him to pay for the printing of photographs, etc.*

*The following contributions will be especially welcome: notes of forthcoming events, meetings held, degrees conferred, staff appointments, honours and public appointments and other local news.*

*Advice on preparation can be obtained from the Editor.*

Printed in Great Britain by John Wright & Sons (Printing) Ltd, at The Stonebridge Press, Bristol

### University of Bristol Faculty of Medicine

### THE DEGREE OF DOCTOR OF PHILOSOPHY
**October 1982**

Claire Amarylis CARTER
Simon Euan NORTHEAST
Roger William PIMLEY
Frabk Gilles Rees TAYLOR
Christopher Vaughan WILLIAMS
Paul Robert WOTTON

### THE DEGREE OF DOCTOR OF MEDICINE

Robert James LUSBY
Ituna Olusola OLUBUYIDE

